# Dietary *Lactobacillus fermentum* and *Bacillus coagulans* Supplementation Modulates Intestinal Immunity and Microbiota of Broiler Chickens Challenged by *Clostridium perfringens*

**DOI:** 10.3389/fvets.2021.680742

**Published:** 2021-05-31

**Authors:** Shuangshuang Guo, Yu Xi, Yi Xia, Tao Wu, Di Zhao, Zhengfan Zhang, Binying Ding

**Affiliations:** Hubei Key Laboratory of Animal Nutrition and Feed Science, School of Animal Science and Nutrition Engineering, Wuhan Polytechnic University, Wuhan, China

**Keywords:** *Lactobacillus fermentum*, *Bacillus coagulans*, *Clostridium perfringens*, immunity, gut microbiota, broiler chicken

## Abstract

Preventative effects of *Lactobacillus fermentum* and *Bacillus coagulans* against *Clostridium perfringens* infection in broilers have been well-demonstrated. The present study was conducted to investigate the modulation of these two probiotics on intestinal immunity and microbiota of *C. perfringens*-challenged birds. The 336 one-day-old broilers were assigned to four groups with six replicates in each group. Birds in the control were unchallenged and fed a basal diet, and birds in the three challenged groups were dietary supplemented with nothing (Cp group), 1 × 10^9^ CFU/kg of *L. fermentum* (Lf_Cp group), or 1 × 10^10^ CFU/kg of *B. coagulans* (Bc_Cp group). Challenge was performed from days 14 to 20, and samples were collected on days 21 and 28. Challenge upregulated interleukin (IL)-1β and transforming growth factor (TGF)-β4 mRNA expression in jejunum on day 21, which was downregulated by *B. coagulans* and *L. fermentum*, respectively (*P* < 0.05). Both probiotic groups upregulated jejunal IL-1β, interferon (IFN)-γ, IL-17, and TGF-β4 on day 28 as well as IFN-γ on day 21 (*P* < 0.05). The Bc_Cp group increased CD3^+^ T cell counts in the jejunal crypt on day 21 (*P* < 0.05). Challenge decreased the ileal ACE index on day 21 and cecal microbial richness on day 28, which were increased by probiotic treatments, and ileal bacterial richness decreased in the Bc_Cp group on day 28 (*P* < 0.05). Only ileal microbiota on day 21 was distinctly affected with an R-value at 0.3116 by ANOSIM analysis (*P* < 0.05). Compared with the control, ileal *Firmicutes* increased on day 21, and ileal *Bacteroidetes* and cecal *Proteobacteria* decreased on day 28 in challenged groups (*P* < 0.05). Challenge increased *Romboutsia* spp. in the ileum as well as unclassified f_*Lachnospiraceae* and *Ruminococcus_torques* group in the cecum, and decreased *Lactobacillus* spp. in the ileum on day 21, which were all conversely modulated by *L. fermentum* (*P* < 0.05). Challenge increased amino acid metabolism of ileal microbiota and membrane transport of cecal microbiota, and decreased amino acid metabolism of cecal microbiota on day 21, which were conversely regulated by both probiotics (*P* < 0.05). In conclusion, *L. fermentum* and *B. coagulans* attenuated the intestinal inflammation and microbial dysbiosis soon after *C. perfringens* challenge.

## Introduction

Necrotic enteritis (NE) is one of the most important enteric diseases in poultry and causes huge economic losses to the industry worldwide (1). Clinical NE is characterized by diarrhea, intestinal necrotic lesions, severe morbidity and mortality, and lower growth rate and feed efficiency, while the subclinical form presents as poor growth performance without mortality (2, 3). *Clostridium perfringens*, the etiological agent of NE, is a spore-forming Gram-positive bacterium and commonly exists in environments such as soil, sewage, litter, feces of chickens, and the intestines of humans and animals (3). The *C. perfringens* population is normally < 10^2^ to 10^4^ colony-forming units (CFU) per gram of intestinal contents in the small intestine of healthy chickens compared to 10^7^ to 10^9^ CFU/g in NE birds (4).

Traditional NE mitigation strategies included antibiotic growth promoters and ionophores. With the increasing pressure to reduce antibiotic use in poultry production, the incidence of NE has been increasing in recent years (5) and alternative nutritional strategies are in urgent need. Probiotics, such as lactic acid bacteria, *Bacillus, Bifidobacteria*, and *Enterococcus*, are potentially alternative strategies to prevent or mitigate NE in birds (6). Probiotics exhibit protective effects by producing antimicrobial compounds, competitive excluding, enhancing the intestinal barrier, and stimulating host immunity (7–9). It has been reported that daily oral administration of *Lactobacillus fermentum* 1.2029 reduced the severity of NE lesions and ameliorated inflammation by upregulating interleukin (IL)-10 mRNA expression and downregulating interferon (IFN)-γ and toll-like receptor (TLR) 2 expression in the ileum of *C. perfringens*-challenged broiler chickens (10). Dietary supplementation with *Bacillus coagulans* decreased gut lesion scores as well as cecal population and hepatic translocation of *C. perfringens*, and increased mRNA expression of fowlicidin-2, an antimicrobial peptide, in the jejunum of NE birds (11).

In recent years, high-throughput sequencing technologies have been used to analyze the bacterial communities in different intestinal segments of chickens (12). Using this technology, Li et al. (13) reported that dietary addition with *L. acidophilus* decreased the Shannon index of ileal microbiota and restored the intestinal microbial community in *C. perfringens*-challenged broiler chickens by increasing relative abundance of beneficial bacteria such as *Lactobacillus* and decreasing abundance of pathogens such as *Escherichia-Shigella*. The NE birds' diet supplemented with *Bacillus subtilis* DSM 32315 had an intestinal microbiota with diversity and composition more similar to that of healthy counterparts reflected by the reduction of opportunistic pathogens and protein-fermentative bacteria (14, 15). However, the effects of *L. fermentum* and *B. coagulans* on the intestinal microbial profiles of NE birds are largely unknown. Therefore, the present study was conducted to investigate the influence of *L. fermentum* and *B. coagulans* on the intestinal immunity and microbiota of broiler chickens infected with *C. perfringens*.

## Materials and Methods

### Experimental Design and Bird Management

All animal procedures used in the present study were performed in compliance with Hubei Provincial Regulations for Laboratory Animals (011043145-029-2013-000009), and were approved by the Institutional Animal Care and Use Committee of Wuhan Polytechnic University. A total of 336 one-day-old sex-mixed broiler chicks were randomly assigned to four groups, each with six replicates of 14 birds. Birds in the control group were fed a corn-soybean meal basal diet and unchallenged by *C. perfringens*. Birds infected with *C. perfringens* were considered as positive control (Cp group). Challenged birds in the Lf_Cp and Bc_Cp groups were fed basal diets supplemented with 1 × 10^9^ CFU/kg of *L. fermentum* and 1 × 10^10^ CFU/kg of *B. coagulans*, respectively. The *L. fermentum* (CGMCC No. 1.2029) was obtained from China General Microbiological Culture Collection Center (CGMCC, Beijing, China) and cultured in Mann-Rogosa-Sharpe broth. The overnight culture of *L. fermentum* was absorbed by nano silicon dioxide and probiotic concentration was 1 × 10^9^ CFU/g of powder. The product of *B. coagulans* (1 × 10^10^ CFU/g) was obtained from Hubei Horwath Biotechnology Co. Ltd. (Wuhan, China). Both *L. fermentum* and *B. coagulans* powder was mixed in the diet at a dose of 0.1%. The basal diet was formulated according to the nutritional requirements of the National Research Council (1994). The composition and nutrient levels of the basal diet are presented in Supplementary Table 1. The trial lasted for 28 days. Birds were raised in wire cages in an environmentally controlled room with 23 h of light and were allowed *ad libitum* access to water and mashed diets throughout the trial.

### *Clostridium perfringens* Challenge

The avian *C. perfringens* type A field strain (CVCC2030) was obtained from China Veterinary Culture Collection Center (Beijing, China). The NE model was established as Du et al. (16) described with modification. The *C. perfringens* was cultured in cooked meat medium and incubated anaerobically overnight at 37°C. The organism was counted using tryptose-sulfite-cycloserine agar plates. From days 14 to 20, challenged birds were orally gavaged with 1 mL of actively growing culture of *C. perfringens* (1 × 10^8^ CFU/mL) once per day. Birds in the control group were gavaged with equal volumes of sterile meat medium.

### Sample Collection

On days 21 and 28, two birds per replicate (12 birds per group) were randomly selected and euthanized by cervical dislocation. Jejunal mucosa was scraped from about 10 cm of the jejunal segment and stored at −80°C for total RNA isolation. Approximately 1 cm of the mid-jejunum was sampled and immediately fixed in 4% paraformaldehyde for immunohistochemical analysis. Digesta from the ileum and cecum was collected and stored at −80°C for DNA extraction.

### RNA Isolation and Quantitative Real-Time PCR

Total RNA of jejunal samples was isolated using Trizol reagent (Invitrogen Life Technologies, Carlsbad, CA, USA) according to the manufacturer's protocol. The concentration and purity of total RNA was quantified by measuring its optical density at 260 and 280 nm with a NanoDrop® ND-2000 UV-VIS spectrophotometer (Thermo Scientific, Wilmington, DE, USA). RNA integrity was verified by agarose gel electrophoresis. One microgram of total RNA was reverse transcribed by the PrimeScript® RT reagent Kit with gDNA Eraser [Takara Biotechnology (Dalian) Co., Ltd., Dalian, China] according to the manufacturer's instructions. The quantitative real-time PCR assay was performed with the 7500 fluorescence detection system (Applied Biosystems, Foster City, CA, USA) according to optimized PCR protocols using the SYBR Premix Ex TaqTM kit [Takara Biotechnology (Dalian) Co., Ltd.]. The primer pairs for the amplification of genes encoding IL-1β, INF-γ, IL-13, IL-17, and transforming growth factor (TGF)-β4 are presented in Supplementary Table 2. β-actin served as an endogenous reference gene. The PCR conditions were an initial denaturation step at 95°C for 30 s, 40 cycles at 95°C for 5 s, annealing and extension temperature at 60°C for 34 s. Each biological sample was run in triplicate. To confirm amplification specificity, the PCR products from each primer pair were subjected to a melting curve analysis and subsequent agarose gel electrophoresis. Gene expression was quantified using the comparative threshold cycle method and the data were expressed as the relative values to the control group.

### Immunohistochemical Analysis

The number of CD3^+^ intraepithelial T cells in the jejunal villus and crypt was determined by immunohistochemical (IHC) analysis as Röhe et al. (17) described with modifications. Briefly, the fixed jejunal tissue was dehydrated and embedded with paraffin wax. The paraffin blocks were consecutively cut at 5 μm and the obtained sections were mounted on glass slides. The indirect immunohistochemical method was used for IHC analysis following the manufacturer's instructions (Boster Biological Technology Co., Ltd., Wuhan, China). After blocking, the jejunal sections were incubated with primary antibody (1:100 dilution) against CD3 T cell receptors overnight at 4°C using humidity chambers. A rat anti-human CD3 antibody, which cross-reacts with the chicken CD3 complex, was used as the primary antibody. Then, tissue sections were incubated with secondary antibody conjugated with horseradish peroxidase (HRP) and CD3^+^ T cells were visualized using a chromogenic dye. Additionally, slides were counterstained with hematoxylin. One of the images of stained slides is presented in Supplementary Figure 1. Finally, the CD3^+^ T cells were quantified using a light microscope (Olympus, Tokyo, Japan), which was equipped with a digital camera (Olympus, Tokyo, Japan) and an image analysis program (ProRes CapturePro software, Jenoptik, Jena, Germany). The data were presented as the number of CD3^+^ T cells per 1,000 μm^2^ in the villus and crypt regions, respectively.

### DNA Extraction

Bacterial DNA from the ileal and cecal digesta was extracted using a QIAamp DNA Stool Mini Kit (Qiagen Inc., Valencia, CA, USA). The DNA concentration was measured with a NanoDrop® ND-2000 UV-VIS spectrophotometer (Thermo Scientific, Wilmington, DE, USA), and the DNA quality was determined by agarose gel electrophoresis. Some samples were pooled together before DNA extraction. Therefore, eight and seven DNA samples in each group were enrolled for high-throughput sequencing on days 21 and 28, respectively.

### Amplification and 16S rRNA Gene Sequencing

The DNA extracted from samples collected on day 21 was amplified and sequenced as Wu et al. (18) described. Briefly, the V3-V4 region of the 16S rRNA gene was amplified by two-stage PCR. The first stage of PCR was performed using the HiFi HotStart ReadyMix kit (Kapa Biosystems, Wilmington, MA, USA) with the forward primer (5′-TCGTCGGCAGCGTCAGATGTGTATAAGAGACAGCCTACGGGNGGCWGCAG-3′) and the reverse primer (5′-GTCTCGTGGGCTCGGAGATGTGTATAAGAGACAGCACTACHVGGGTATCTAATCC-3′). The second stage of index PCR was processed using the Nexteta XT Index kit (Illumina, San Diego, CA, USA), in which the dual indices and Illumina sequencing adapters were attached. Then, PCR products were purified with the Agencourt AMPure XP purification system (Beckman, Brea, CA, USA) and quantified by the SpectraMax i3X Multi-mode detection platform (Molecular Devices, San Jose, CA, USA). The purified amplicons were sequenced using the Illumina MiSeq platform (San Diego, CA, USA).

Due to technical issues, the DNA amplification and bacterial 16S rRNA gene sequencing for samples collected on day 28 was performed by Shanghai Majorbio Bio-Pharm Technology Co., Ltd. (Shanghai, China). The V3-V4 variable region of 16S RNA gene was amplified with barcoded primer pair 338F/806R (338F: 5′-ACTCCTACGGGAGGCAGCAG-3′; 806R: GGACTACHVGGGTWTCTAAT-3′). The amplicons were then extracted from 2% agarose gels and purified using the AxyPrep DNA Gel Extraction Kit (Axygen Biosciences, Union City, CA, USA) and quantified by a Quantus^TM^ Fluorometer (Promega Corporation, Madison, WI, USA). Purified amplicons were pooled in equimolar amounts and pair-end sequenced (2 × 300) on an Illumina MiSeq platform (San Diego, CA, USA).

### Bioinformatics Analysis

Bioinformatics analysis was performed using the Majorbio Cloud Platform (http://www.majorbio.com). Raw sequencing reads were demultiplexed and quality-filtered by Trimmomatic software (version 0.36) and then merged by FLASH software (version 1.2.7). Operational taxonomic units (OTUs) were clustered with the 97% similarity cut-off using UPARSE software (version 7.1). The taxonomy of each OTU representative sequence was analyzed using the RDP Classifier (http://rdp.cme.msu.edu/) against the SILVA rRNA database (http://www.arb-silva.de) with a confidence threshold of 70%. After the elimination of interference sequence, a rarefaction curve and Venn diagram were created using R software (version 2.15.3). Analysis of alpha diversity was performed using Mothur software (version 1.30.1). Beta diversity was evaluated by the unweighted Unifrac principal co-ordinates analysis (PCoA) and the significance of bacterial separation among groups was reflected by ANOSIM analysis. Both analyses were conducted using R software. The top 10 differentially abundant bacteria at the genus level in the control vs. Cp group, Cp vs. Lf_Cp group, and Cp vs. Bc_Cp group were analyzed with Student's *t*-test and a *P* value threshold of 0.05 was used. PICRUSt analysis was conducted to predict the potential function of microbiota. The OTUs were normalized by copy number and metagenome prediction was categorized into the Kyoto Encyclopedia of Genes and Genomes (KEGG) at level 2 (19).

### Statistical Analysis

The data of intestinal immune indices, bacterial alpha diversity, top 4 phyla, and top 5 predicted KEGG pathways were analyzed by one-way ANOVA using SPSS version 21.0 (SPSS Inc., Chicago, IL, USA). When significant differences were observed, individual group means were compared using Duncan's multiple comparison. If data did not comply with normal distribution, which was analyzed by a Kolmogorov-Smirnov test, a non-parameter test and pairwise comparisons were used to analyze the data. Data in figures are presented as mean and SE, while data in tables are given as mean and pooled SEM. *P* < 0.05 was considered statistically significant.

## Results

### Jejunal Cytokine Gene Expression

**Figure 1:**
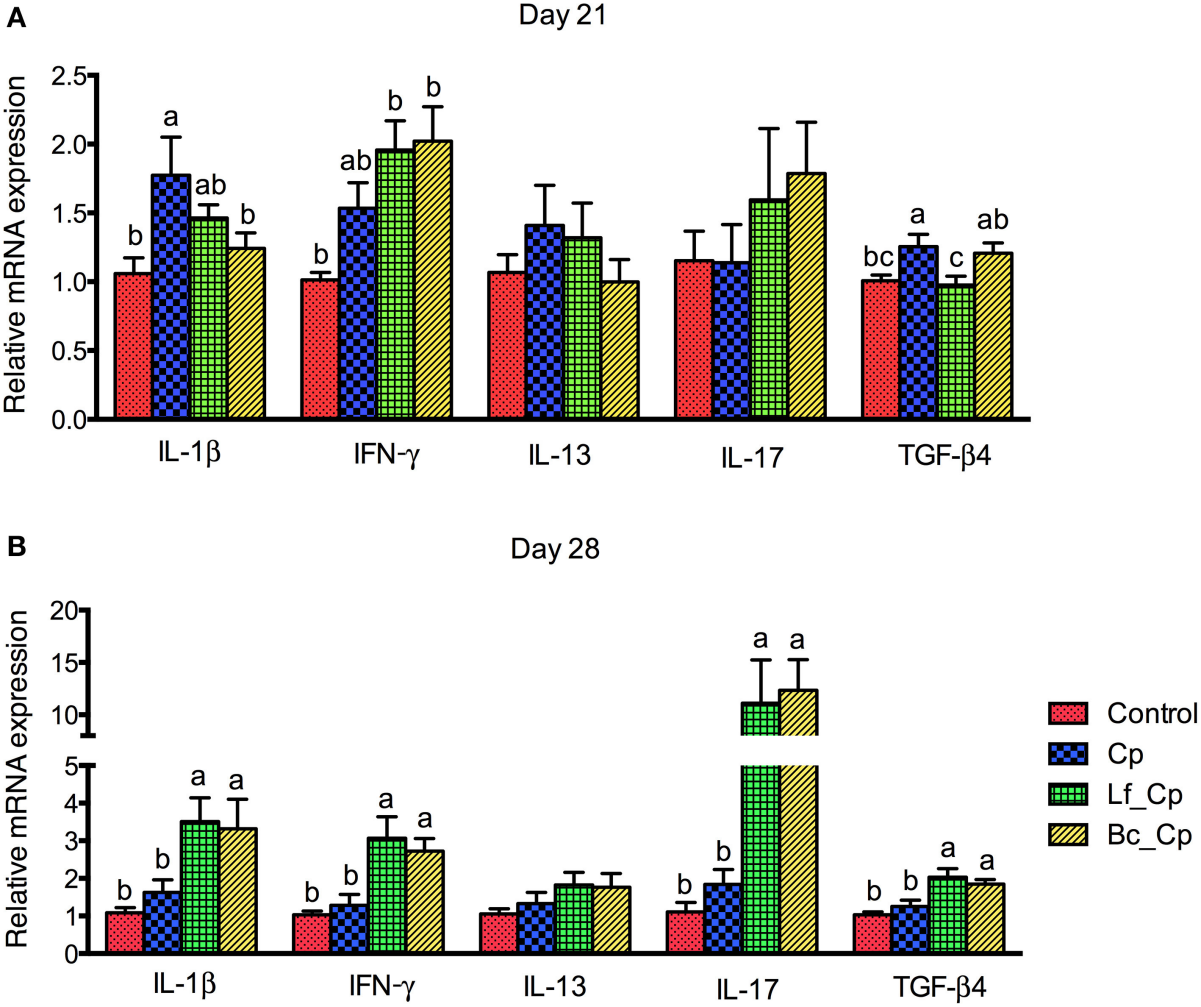
The cytokine mRNA expression in jejunal mucosa of *Clostridium perfringens*-challenged broiler chickens fed diets supplemented with *Lactobacillus fermentum* or *Bacillus coagulans* on days 21 **(A)** and 28 **(B)**. Data were expressed as mean and SE from 12 chickens. Control, unchallenged group; Cp, *C. perfringens*-challenged group; Lf_Cp, challenged group with dietary supplementation of *Lactobacillus fermentum*; Bc_Cp, challenged group with dietary supplementation of *Bacillus coagulans*. IL, interleukin; IFN, interferon; TGF, transforming growth factor.

The mRNA expression of cytokines in the jejunum is revealed in Figure 1. At 21 days of age, compared with the control group, the Cp group upregulated the mRNA expression of IL-1β and TGF-β4 (Figure 1A, *P* < 0.05). However, compared with the Cp group, the Bc_Cp and Lf_Cp groups downregulated the IL-1β and TGF-β4 mRNA expression, respectively (*P* < 0.05). The gene expression of IL-13 and IL-17 was not significantly affected (*P* < 0.05). At 28 days of age, compared with the control and Cp groups, the Lf_Cp and Bc_Cp groups significantly upregulated the mRNA expression of IL-1β, INF-γ, IL-17, and TGF-β4 in the jejunum (Figure 1B, *P* < 0.05). The mRNA level of IL-13 was not influenced by challenge and dietary treatments (*P* > 0.05).

### Jejunal CD3^+^ T Cell Counts in Jejunum

**Figure 2:**
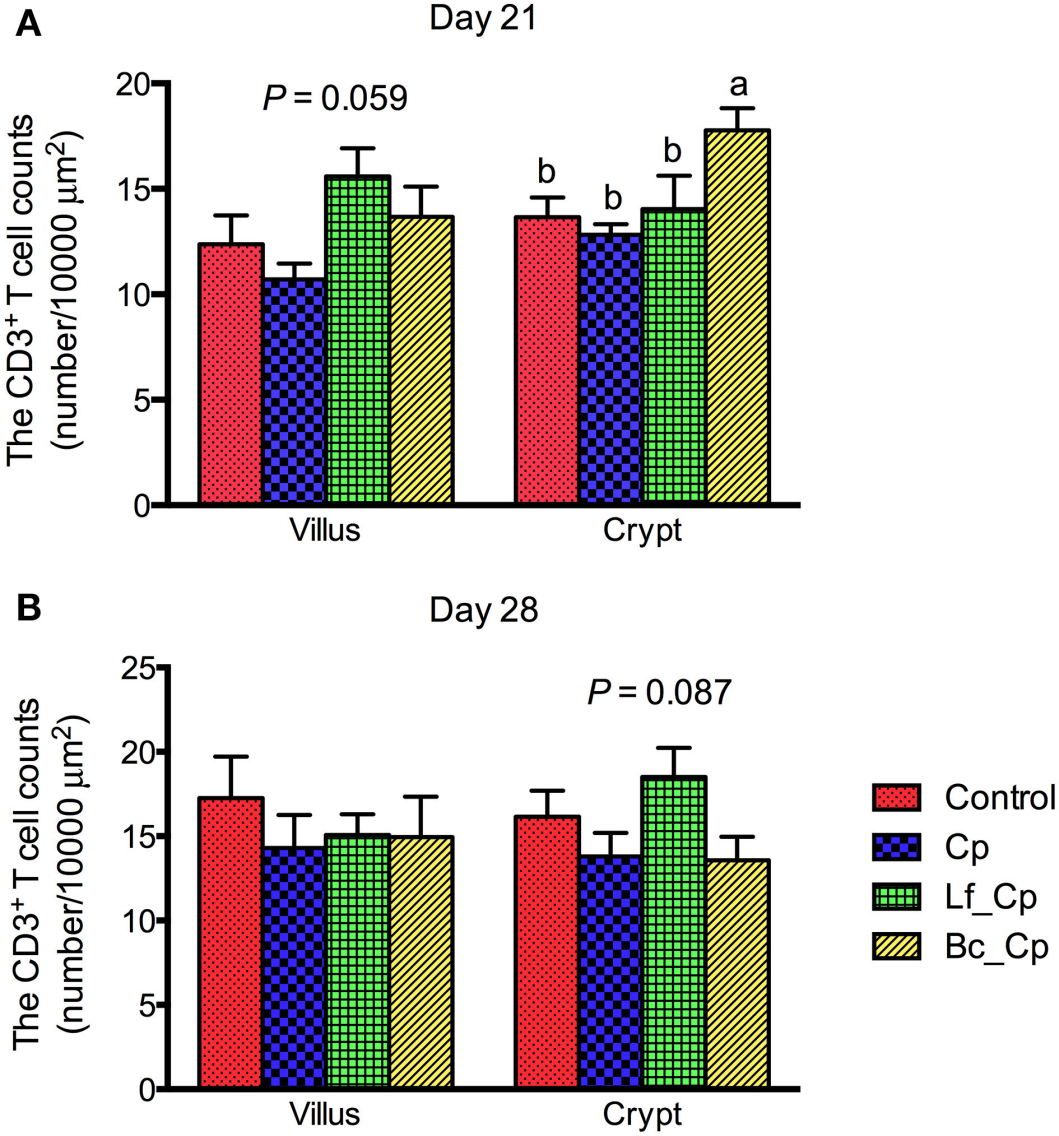
The CD3^+^ T cell counts in villus and crypt of jejunum of *Clostridium perfringens*-challenged broiler chickens fed diets supplemented with *Lactobacillus fermentum* or *Bacillus coagulans* on days 21 **(A)** and 28 **(B)**. Data were expressed as mean and SE from 12 chickens. Control, unchallenged group; Cp, *C. perfringens*-challenged group; Lf_Cp, challenged group with dietary supplementation of *Lactobacillus fermentum*; Bc_Cp, challenged group with dietary supplementation of *Bacillus coagulans*.

The CD3^+^ T cell counts in the villus and crypts of the jejunum are presented in Figure 2. At 21 days of age, the Lf_Cp group tended to increase the villus CD3^+^ T cell counts in contrast to the Cp group (Figure 2A, *P* = 0.059). The Bc_Cp group had higher crypt CD3^+^ T cell counts than that of the other three groups (*P* < 0.05), among which the cell count did not significantly differ (*P* > 0.05). At 28 days of age, the jejunal CD3^+^ cell counts were not significantly affected by treatments (Figure 2B, *P* > 0.05). The Lf_Cp group tended to have a higher CD3^+^ T cell number in the crypt of the jejunum than that of the Cp and Bc_Cp groups (*P* = 0.087).

### Quality of Sequencing Data and Venn Diagram

The sequencing of ileal and cecal samples collected on days 21 and 28 generated a total of 3,215,602, 4,590,839, 1,482,180, and 1,624,227 effective sequences, respectively (Supplementary Tables 3, 4). The median read length for these samples was 451, 449, 433, and 433 base pairs, respectively. The Good's coverage (Supplementary Tables 3, 4) and rarefaction curves (Supplementary Figure 2) showed that sufficient sequencing coverage for all samples was achieved. All treatments shared 270 and 400 OTUs in ileal and cecal microbial communities at 21 days of age, respectively (Supplementary Figures 3A,C). At 28 days of age, there were 226 and 406 common OTUs in ileal and cecal microbiota of the four groups, respectively (Supplementary Figures 3B,D).

### Alpha Diversity of Microbiota in Ileum and Cecum

As seen in Table 1, at 21 days of age, compared with the control group, the Cp group tended to decrease Sobs (*P* = 0.087) and Chao1 (*P* = 0.096) indices and significantly reduced the ACE index of ileal microbiota (*P* < 0.05). Meanwhile, the Bc_Cp group had comparable Sobs and ACE indices to the control. The alpha diversity of cecal microbiota was not significantly affected by treatments on day 21. At 28 days of age, the Bc_Cp group had lower richness indices (Sobs, Chao1, and ACE) than those of the other three groups in ileal microbiota (*P* < 0.05). Compared with the Cp group, the Lf_Cp group tended to increase the Shannon index (*P* = 0.055) and decrease the Simpson index of ileal microbiota (*P* = 0.087). In the cecum, the Cp group decreased in richness indices (Chao1 and ACE) in contrast to the control and Bc_Cp groups (*P* < 0.05). The Cp group tended to have lower Sobs (*P* = 0.075) and Shannon (*P* = 0.058) indices of cecal microbiota than those in the other three groups.

**Table 1:** The alpha diversity of ileal and cecal microbiota in broiler chickens^1^.

**Item**	**Shannon**	**Simpson**	**Sobs**	**Chao 1**	**ACE**
Day 21					
Ileum					
Control	2.59	0.22	233.38	276.68	289.09^a^
Cp	1.70	0.44	158.75	193.15	191.64^b^
Lf_Cp	1.78	0.35	187.88	230.31	236.10^a^^b^
Bc_Cp	2.55	0.29	228.13	263.77	270.76^a^
SEM	0.17	0.04	12.09	13.01	12.62
*P*-value	0.122	0.192	0.087	0.096	0.025
Cecum					
Control	3.26	0.15	297.50	367.41	371.15
Cp	3.38	0.13	286.50	362.88	363.66
Lf_Cp	3.21	0.16	314.88	384.51	381.43
Bc_Cp	3.12	0.16	276.38	344.71	336.79
SEM	0.06	0.01	5.50	8.04	7.32
*P*-value	0.447	0.578	0.073	0.390	0.164
Day 28					
Ileum					
Control	1.87	0.37	281.00^a^	307.65^a^	308.70^a^
Cp	1.47	0.43	208.14^a^	248.64^a^	261.43^a^
Lf_Cp	2.44	0.20	229.71^a^	247.86^a^	248.96^a^
Bc_Cp	1.65	0.33	114.00^b^	138.36^b^	147.88^b^
SEM	0.14	0.03	18.37	18.63	17.84
*P*-value	0.055	0.087	0.006	0.006	0.006
Cecum					
Control	3.39	0.11	347.00^a^	372.62^a^	367.82^a^
Cp	2.66	0.19	263.29^b^	289.83^b^	285.92^b^
Lf_Cp	3.33	0.14	321.00^a^	341.32^a^^b^	336.98^a^^b^
Bc_Cp	3.50	0.11	349.14^a^	370.23^a^	366.51^a^
SEM	0.12	0.02	10.99	11.09	10.44
*P*-value	0.058	0.268	0.011	0.019	0.009

1 *Data are expressed as mean and pooled SEM from 8 and 7 chickens on days 21 and 28, respectively*.

a, b *Values within a row with different superscripts differ significantly at P < 0.05. Control, unchallenged group; Cp, Clostridium perfringens-challenged group; Lf_Cp, challenged group with dietary supplementation of Lactobacillus fermentum; Bc_Cp, challenged group with dietary supplementation of Bacillus coagulans*.

### Beta Diversity of Microbiota in Ileum and Cecum

**Figure 3:**
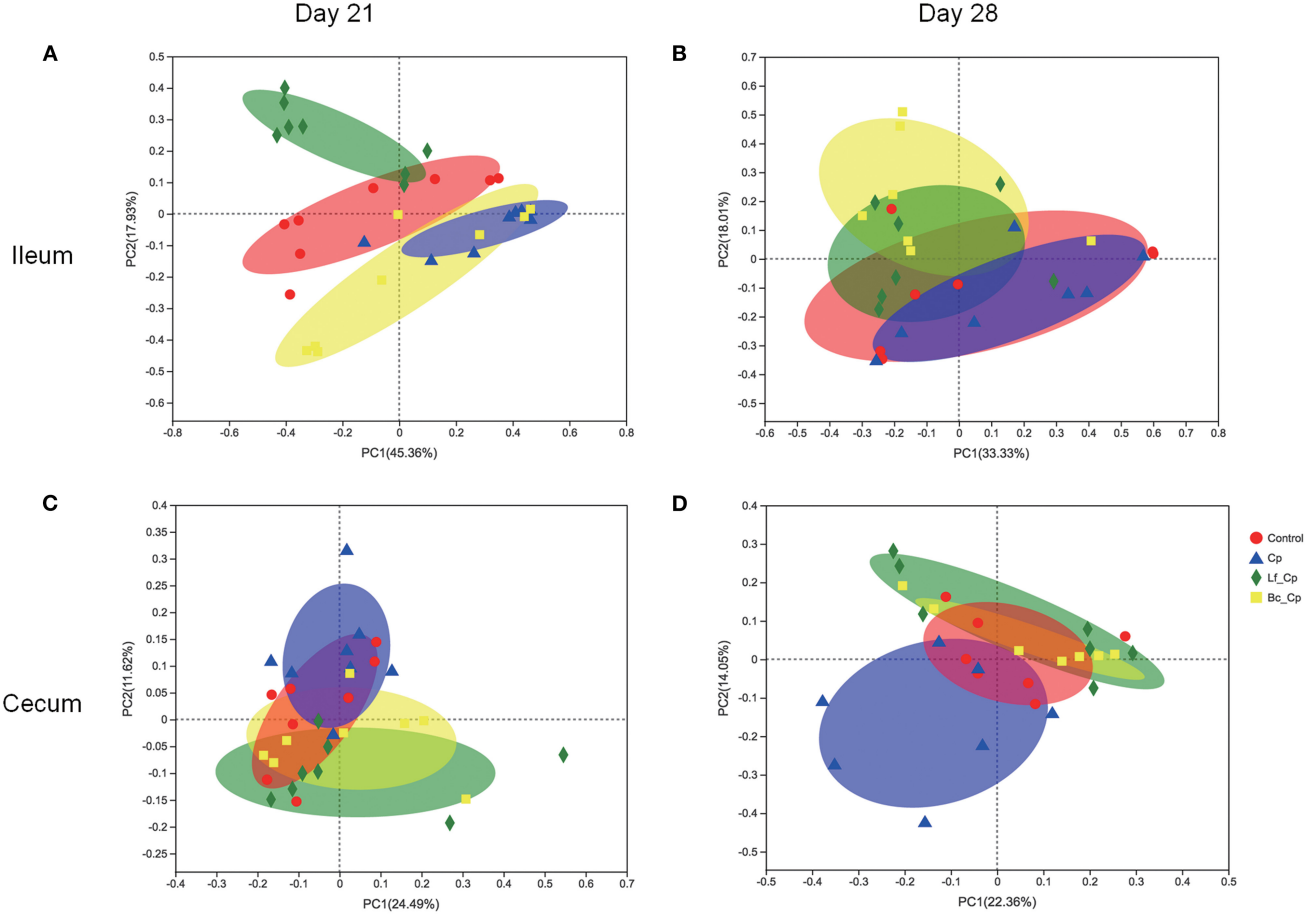
The PCoA plots of intestinal microbiota of *Clostridium perfringens*-challenged broiler chickens fed diets supplemented with *Lactobacillus fermentum* or *Bacillus coagulans* on days 21 **(A,C)** and 28 **(B,D)**. Data were derived from 8 and 7 chickens on days 21 and 28, respectively. Control, unchallenged group; Cp, *C. perfringens*-challenged group; Lf_Cp, challenged group with dietary supplementation of *Lactobacillus fermentum*; Bc_Cp, challenged group with dietary supplementation of *Bacillus coagulans*.

Beta diversity illustrated via PCoA is presented in Figure 3. At 21 days of age, the Lf_Cp group had distinct ileal microbiota compared with the other three groups, with a slight similarity with the control (Figure 3A). Meanwhile, ileal microbiota on day 21 in the Cp group was much similar to that in the Bc_Cp group. Cecal microbiota on day 21 as well as ileal and cecal microbiota on day 28 was not notably differentiated by treatments. However, the positive R-values in ANOSIM analysis suggested that the inter-group difference of microbial communities in both the ileum and cecum at two time points was greater than the intra-group difference (Table 2, *P* < 0.05).

**Table 2:** Similarities of bacterial composition among groups by ANOSIM analysis^a^.

**Item**	***R*-value**	***P*-value**
d 21		
Ileum	0.3116	0.002
Cecum	0.1085	0.010
d 28		
Ileum	0.1516	0.021
Cecum	0.1539	0.008

a *Data are derived from 8 and 7 chickens on days 21 and 28, respectively*.

### Bacterial Composition at Phylum Level in Ileum and Cecum

The ileal and cecal bacterial composition at the phylum level is shown in Supplementary Figure 4. The top four phyla in each intestinal segment and at each time point are presented in Table 3. At 21 days of age, compared with the control, the Cp and Lf_Cp groups increased the relative abundance of *Firmicutes* in the ileum (*P* < 0.05). The bacterial compositions at the phylum level in the cecum were not significantly affected by treatments (*P* > 0.05). At 28 days of age, compared with the control, the Cp and Bc_Cp groups decreased in ileal *Bacteroidetes* abundance, and all other groups reduced in cecal *Proteobacteria* abundance (*P* < 0.05).

**Table 3:** The top 4 phyla in the ileal and cecal microbiota of broiler chickens^1^.

**Item**	**Control**	**Cp**	**Lf_Cp**	**Bc_Cp**	**SEM**	***P*-value**
Day 21						
Ileum						
*Firmicutes*	72.66^b^	90.87^a^	92.06^a^	77.04^a^^b^	3.09	0.049
*Bacteroidetes*	9.30	3.61	2.62	11.79	1.45	0.232
*Proteobacteria*	7.80	2.55	2.73	5.02	0.87	0.107
*Cyanobacteria*	8.74	2.12	1.75	3.34	1.22	0.612
Cecum						
*Firmicutes*	58.79	63.94	62.32	62.50	1.74	0.775
*Bacteroidetes*	36.85	32.30	34.78	32.74	1.86	0.830
*Verrucomicrobia*	3.04	2.30	1.94	3.09	0.42	0.740
*Proteobacteria*	1.18	1.22	0.76	1.50	0.22	0.719
Day 28						
Ileum						
*Firmicutes*	39.26	49.47	45.19	46.23	5.09	0.923
*Cyanobacteria*	36.87	40.79	31.50	26.18	4.66	0.727
*Proteobacteria*	16.20	8.78	16.44	26.10	3.27	0.329
*Bacteroidetes*	6.47^a^	0.69^b^	4.76^a^^b^	1.19^b^	1.08	0.023
Cecum						
*Firmicutes*	59.58	62.86	62.83	66.40	2.66	0.860
*Bacteroidetes*	29.09	33.12	32.35	27.70	2.62	0.879
*Proteobacteria*	9.47^a^	1.69^b^	2.86^b^	4.61^b^	0.90	0.005
*Tenericutes*	0.85	0.38	1.28	1.02	0.17	0.285

1 *Data are expressed as mean and pooled SEM from 8 and 7 chickens on days 21 and 28, respectively*.

a, b *Values within a row with different superscripts differ significantly at P < 0.05. Control, unchallenged group; Cp, Clostridium perfringens-challenged group; Lf_Cp, challenged group with dietary supplementation of Lactobacillus fermentum; Bc_Cp, challenged group with dietary supplementation of Bacillus coagulans*.

### Top 10 Differentially Abundant Bacteria at Genus level in Ileum and Cecum

**Figure 4:**
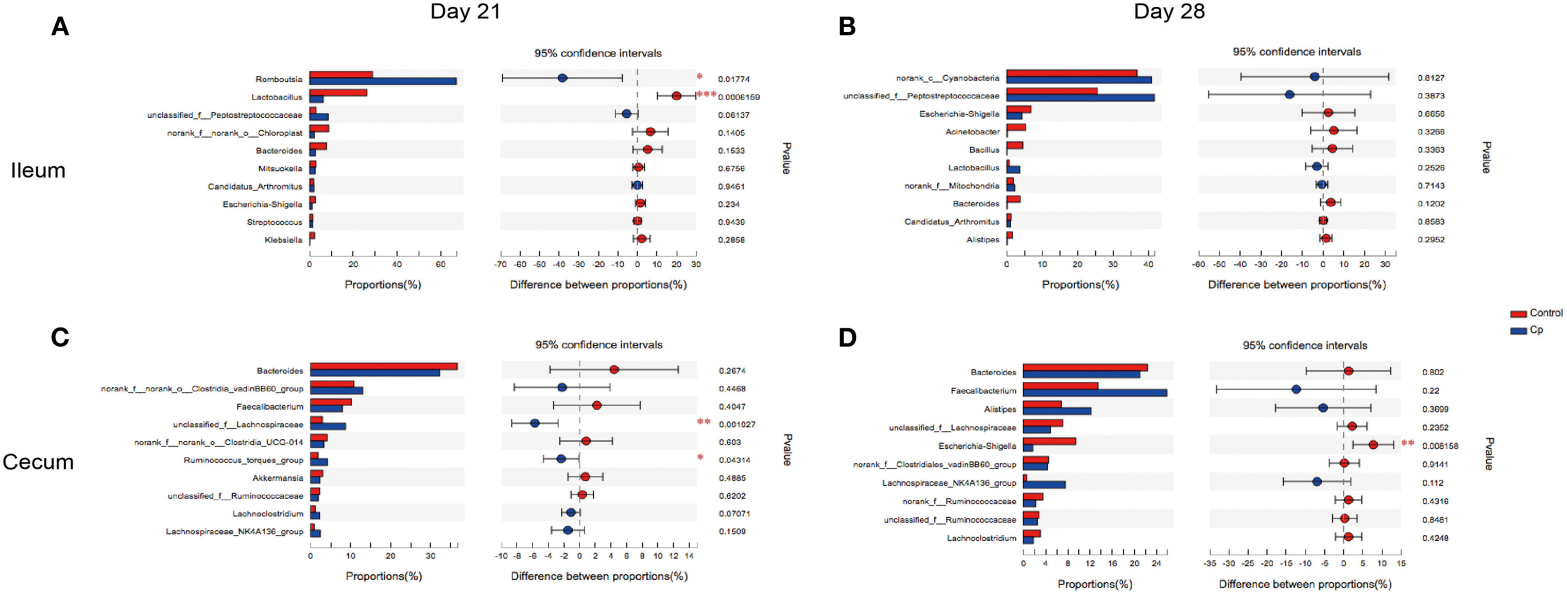
The top 10 differentially abundant bacteria at genus level in control vs. Cp groups. Ileal and cecal microflora on days 21 **(A,C)** and 28 **(B,D)** was analyzed. Data were derived from 8 and 7 chickens on days 21 and 28, respectively. Control, unchallenged group; Cp, *C. perfringens*-challenged group. *P < 0.05, **P < 0.05, ***P < 0.01.

**Figure 5:**
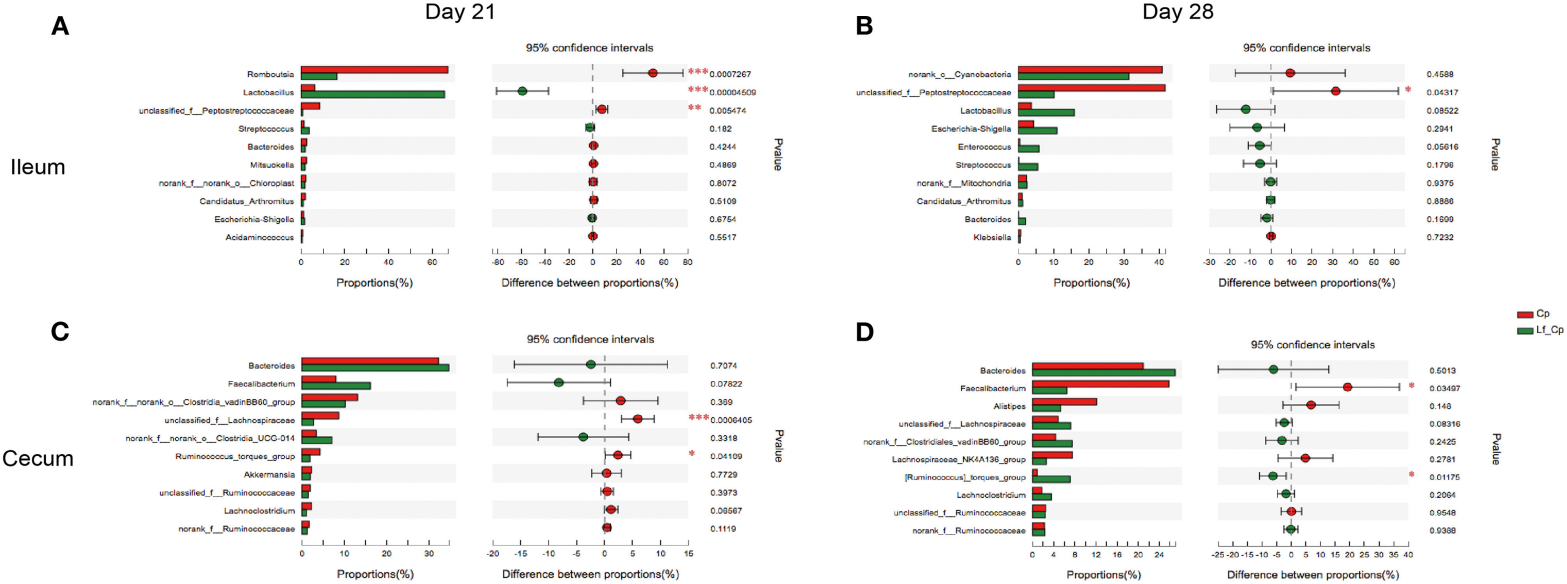
The top 10 differentially abundant bacteria at genus level in Cp vs. Lf_Cp groups. Ileal and cecal microflora on days 21 **(A,C)** and 28 **(B,D)** was analyzed. Data were derived from 8 and 7 chickens on days 21 and 28, respectively. Cp, *C. perfringens*-challenged group; Lf_Cp, challenged group with dietary supplementation of *Lactobacillus fermentum*. *P < 0.05, **P < 0.05, ***P < 0.01.

**Figure 6:**
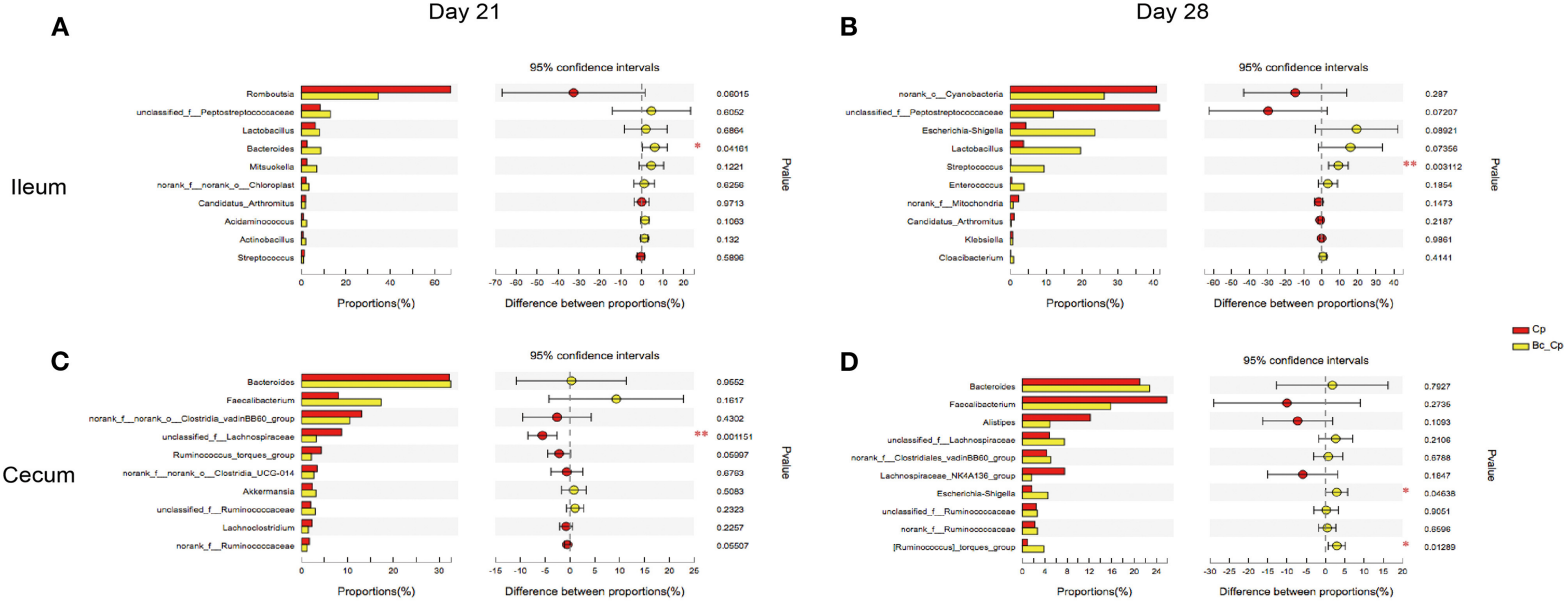
The top 10 differentially abundant bacteria at genus level in Cp vs. Bc_Cp groups. Ileal and cecal microflora on days 21 **(A,C)** and 28 **(B,D)** was analyzed. Data were derived from 8 and 7 chickens on days 21 and 28, respectively. Cp, *C. perfringens*-challenged group; Bc_Cp, challenged group with dietary supplementation of *Bacillus coagulans*. *P < 0.05, **P < 0.05.

The top 10 differentially abundant genera in selected groups are seen in Figures 4–**6**.

At 21 days of age, compared with the control, the Cp group increased the relative abundance of *Romboutsia* spp. and decreased in *Lactobacillus* spp. abundance in the ileum (Figure 4A, *P* < 0.05). Compared with the Cp group, the Lf_Cp group decreased the relative contents of *Romboutsia* spp. and unclassified f_*Peptostreptococcaceae*, and increased the *Lactobacillus* spp. abundance in the ileum (Figure 5A, *P* < 0.05). The Bc_Cp group had a higher ileal *Bacteroides* spp. content than that of the Cp group (Figure 6A, *P* < 0.05). In contrast to the control, the Cp group increased unclassified f_*Lachnospiraceae* and *Ruminococcus_torques* group abundance in the cecum (Figure 4C, *P* < 0.05), both of which was decreased in the Lf_Cp group (Figure 5C, *P* < 0.05). Compared with the Cp group, the Bc_Cp group decreased cecal unclassified f_*Lachnospiraceae* content (Figure 6C, *P* < 0.05).

At 28 days of age, *C. perfringens* challenge did not induce significant changes in ileal microbiota (Figure 4B, *P* > 0.05). Compared with the Cp group, the Lf_Cp group decreased in the relative abundance of unclassified f_*Peptostreptococcaceae* in the ileum (Figure 5B, *P* < 0.05). Meanwhile, the Bc_Cp group increased ileal *Streptococcus* spp. content (Figure 6B, *P* < 0.05). In the cecum, *C. perfringens* challenge decreased the relative abundance of *Escherichia-Shigella* (Figure 4D, *P* < 0.05). Compared with the Cp group, the Lf_Cp group reduced the relative content of *Faecalibacterium* spp. and increased *Ruminococcus*_torques group content in the cecum (Figure 5D, *P* < 0.05). The Bc_Cp group increased the relative abundance of both *Escherichia-Shigella* and *Ruminococcus*_torques group in the cecum (Figure 6D, *P* < 0.05).

### Predicted Functional Potentials of OTUs in Ileum and Cecum

The top five presumptive functions of microbiota at level 2 of the KEGG pathways are illustrated in Table 4. At 21 days of age, the Lf_Cp group had a higher abundance of the replication and repair pathway and translation pathway than that of the other three groups in the ileum (*P* < 0.05). Compared with the control, the Cp group increased ileal abundance of amino acid metabolism, but the Lf_Cp group decreased this pathway abundance (*P* < 0.05). In contrast to the control, the Cp group increased the abundance of the membrane transport pathway, but decreased amino acid metabolism in the cecum (*P* < 0.05). Both probiotic-supplemented groups exhibited opposite changes to the Cp group (*P* < 0.05) and did not significantly differ from the control (*P* > 0.05). The Lf_Cp group had higher abundance of the replication and repair pathway than that of the control in the cecum (*P* < 0.05). At 28 days of age, compared with the control and Cp groups, the Bc_Cp and Lf_Cp groups increased the abundance of ileal membrane transport and cecal carbohydrate metabolism, respectively (*P* < 0.05).

**Table 4:** The top 5 abundant microbial pathway grouped into level 2 functional categories using PICRUSt^1^.

**Item**	**Control**	**Cp**	**Lf_Cp**	**Bc_Cp**	**SEM**	***P*-value**
Day 21						
Ileum						
Membrane transport	13.45	13.56	13.81	13.03	0.24	0.722
Carbohydrate metabolism	10.18	10.12	10.37	10.39	0.11	0.789
Replication and repair	8.86^b^	8.71^b^	11.06^a^	8.65^b^	0.23	<0.001
Amino acid metabolism	8.74^b^	9.55^a^	7.34^c^	9.36^a^^b^	0.19	<0.001
Translation	5.44^b^	5.28^b^	6.81^a^	5.31^b^	0.14	<0.001
Cecum						
Membrane transport	11.40^b^	12.82^a^	11.61^b^	11.74^b^	0.15	0.001
Carbohydrate metabolism	10.89	10.90	10.82	10.83	0.03	0.689
Amino acid metabolism	9.75^a^	9.55^b^	9.73^a^	9.71^a^	0.02	0.002
Replication and repair	8.78^a^^b^	8.62^b^	8.94^a^	8.80^a^^b^	0.04	0.011
Energy metabolism	5.90	5.82	5.91	5.87	0.01	0.097
Day 28						
Ileum						
Membrane transport	11.69^b^	11.79^b^	12.16^b^	12.91^a^	0.15	0.011
Carbohydrate metabolism	9.02	8.79	9.17	9.30	0.09	0.253
Amino acid metabolism	8.97	8.85	8.45	8.27	0.11	0.069
Replication and repair	8.04	8.14	8.31	8.19	0.10	0.853
Energy metabolism	8.11	8.39	8.17	7.60	0.28	0.803
Cecum						
Membrane transport	12.35	11.72	12.27	12.71	0.22	0.487
Carbohydrate metabolism	10.88^b^	11.00^b^	11.25^a^	11.06^a^^b^	0.05	0.025
Amino acid metabolism	9.58	9.81	9.66	9.57	0.04	0.069
Replication and repair	8.76	8.94	8.77	8.73	0.05	0.526
Energy metabolism	5.79	5.84	5.86	5.81	0.02	0.688

* *Data are expressed as mean and pooled SEM from 8 and 7 chickens on days 21 and 28, respectively*.

a−c *Values within a row with different superscripts differ significantly at P < 0.05. Control, unchallenged group; Cp, Clostridium perfringens-challenged group; Lf_Cp, challenged group with dietary supplementation of Lactobacillus fermentum; Bc_Cp, challenged group with dietary supplementation of Bacillus coagulans*.

## Discussion

The previously reported results from the current study showed that the *C. perfringens* challenge-induced NE model was successful, evidenced by decreased averaged feed intake and increased jejunal lesion score as well as elevated *C. perfringens* counts in the ileum and cecum (20). Furthermore, both *L. fermentum* and *B. coagulans* addition inhibited intestinal pathogen colonization and *B. coagulans* exhibited superior effects in alleviating intestinal injury (20). Therefore, the intestinal immunity and microbiota of *C. perfringens*-challenged birds fed *L. fermentum* and *B. coagulans*-supplemented diets were further investigated in the present study.

The *in vivo* and *in vitro* studies have demonstrated that *C. perfringens* challenge evoked intense intestinal inflammatory responses *via* TLRs or nucleotide-binding oligomerization domains triggered the nuclear factor kappa B signaling pathway in broilers (9, 21). This signaling cascade resulted in the activation of macrophages and/or dendritic cells, which in turn led to the differentiation of naïve T helper (Th) cells into mature effector Th1, Th2, Th17, and Treg cells, which characteristically produced IL-1, INF-γ, IL-13, IL-17, and TGF-β, respectively (22, 23). Fasina and Lillehoj (24) reported that *C. perfringens* infection induced intestinal inflammation via the activation of Th2 and Th17 cells and inhibition of Treg cells, proven by the upregulation of IL-13 and IL-17 and downregulation of TGF-β4. In the present study, *C. perfringens* challenge upregulated jejunal IL-1β and TGF-β4 expression, which was downregulated by *B. coagulans* and *L. fermentum*, respectively, soon after the infection (day 21). However, both probiotics increased the mRNA expression of IL-1β, INF-γ, IL-13, IL-17, and TGF-β in the jejunum at seven days post infection (day 28). This suggested that probiotics showed inhibitory and stimulatory effects on cytokine expression of Th cells in the acute and recovery phase of *C. perfringens* challenge, respectively. Meanwhile, they mainly modulated the functions of Th1, Th17, and Treg cells. However, Emami et al. (25) reported that *Bacillus licheniformis* alone or combined with prebiotic and essential oil downregulated the IFN-γ, IL-10, and IL-17 mRNA abundance in the jejunum of broilers with subclinical NE, indicating a inhibition of Th2, Th17, and Treg cell function.

Intraepithelial lymphocytes (IEL), consisting of natural killer cells, T cells, and B cells, are an important component in gut-associated lymphoid tissue, which is one of the major immunological systems in chickens (26). Increase of the general T cell (CD3^+^) population within the intestinal epithelium indicates a strong local immune system with increased resistance to enteric diseases (27). In the present study, *L. fermentum* tended to increase CD3^+^ T cell density in the villus on day 21 and in the crypt on day 28, and *B. coagulans* significantly elevated crypt CD3^+^ T cell population in the jejunum of *C. perfringens*-challenged broilers. This suggested that *L. fermentum* and *B. coagulans* addition might induce intestinal T cell responses to fight against *C. perfringens* infection in broiler chickens. Consistently, Bai et al. (28) observed that *L. fermentum* JS combined with *Saccharomyces cerevisiae* stimulated the intestinal T cell immune system by increasing the subpopulations of IEL CD3^+^, CD4^+^, and CD8^+^ T cells in the jejunum of broiler chickens. It was reported that *B. subtilis* spores as adjuvants with inactivated H9N2 enhanced CD4^+^ and CD8^+^ T cell responses in White Leghorn chickens against avian influenza H9N2 (29).

It has been demonstrated that the diversity of microbial community contributed to the homeostasis of intestinal microbiota and the resistance to pathogens (30). In the present study, *C. perfringens* challenge decreased the richness (Sobs, Chao1, and ACE indices) and diversity (Shannon index) of cecal microbiota at 28 days of age, and both probiotic groups had comparable alpha diversity to the control. The same change of ACE index was seen in the ileal microbiota at 21 days of age. This indicated that the impaired intestinal microbial richness and diversity was restored by probiotic addition. Partly consistent with our findings, Li et al. (13) reported that *C. perfringens* infection decreased the richness of ileal bacterial community of broiler chickens and dietary *L. acidophilus* addition reduced its diversity, but both challenge and probiotic supplementation increased the richness and diversity of cecal microbiota. Furthermore, we found that *B. coagulans* significantly decreased ileal bacterial richness at 28 days of age. In a non-challenged model, broilers fed *B. coagulans* TBC169 had higher diversity of jejunal microbiota than that of the control on day 21, but lower diversity on day 42, although both of which did not achieve statistical significance (31).

In the current study, beta diversity analysis indicated only ileal microbiota at 21 days of age was markedly altered by treatments with the highest R-value (0.3116) by ANOSIM analysis. The unchallenged and challenged birds without probiotic addition had distinct microbiota only in the ileum on day 21. Compared with the *C. perfringens*-challenged group, *L. fermentum* alone and both probiotics altered intestinal microbiota on days 21 and 28, respectively. Necrotic enteritis in broilers was caused by *C. perfringens* proliferation and manifested by lesions in the small intestine (1). Moreover, the exogenous *C. perfringens* administration could not totally colonize in the intestine and they might be gradually excreted after challenge. This was evidenced by our previous observation that the relative abundance of *C. perfringens* in ileal and cecal contents quantified by qPCR in the challenged group without probiotic supplementation was much higher than that in the control on day 21 and no significant *C. perfringens* abundance was seen on day 28 (20). The influence of probiotic addition on the intestinal microbiota of birds challenged by *C. perfringens* might be disclosed in the following description.

It has been reported that *Firmicutes, Cyanobacteria, Proteobacteria*, and *Bacteroidetes* were the four predominant bacterial phyla in ileal microbiota (19, 32), and *Firmicutes, Proteobacteria, Bacteroidetes, Tenericutes*, and *Verrucomicrobia* were the predominant phyla in cecal microbiota (13, 33). This was consistent with our findings. Meanwhile, challenged birds in the Cp and Lf_Cp groups had higher relative abundance of ileal *Firmicutes* than that of birds in the control group on day 21, and challenged birds had lower ileal *Bacteroidetes* and cecal *Proteobacteria* abundance on day 28. Contrary to our observation, Xu et al. (34) demonstrated that NE severity was related to a decrease of *Firmicutes* and an increase of *Bacteroidetes* and *Proteobacteria*. Moreover, Zhang et al. (19) observed that *C. perfringens* challenge significantly decreased ileal *Firmicutes* abundance in broilers. The increase of *Firmicutes* population could suppress *C. perfringens* and restore intestinal homeostasis (35). It was speculated that the increase of *Firmicutes* abundance in the Cp and Lf_Cp groups might be a strategy for the host to maintain ileal microbial homeostasis. The increase of *Bacteroidetes* was associated with a decrease of nutrient absorption (36). *Proteobacteria* contain a wide variety of pathogens such as *Escherichia coli, Salmonella*, and *Shigella*, which can colonize in the intestine of chickens (37). The decrease of *Proteobacteria* and *Bacteroidetes* in challenged birds on day 28 might indicate that a healthy bacterial community was achieved in the recovery phase post challenge.

At 21 days of age, *C. perfringens* challenge increased *Romboutsia* spp. in the ileum as well as unclassified f_*Lachnospiraceae* and *Ruminococcus_torques* group in the cecum, and decreased *Lactobacillus* spp. in the ileum, which were all conversely modulated by dietary *L. fermentum* addition. *Romboutsia* spp. was reported to be associated with less severe immune responses accompanied with decreasing levels of pro-inflammatory cytokines in plasma (38). It was predominant in the feces of nursing home residents with *Clostridium difficile* colonization (39). Therefore, the decrease of *Romboutsia* spp. abundance in challenged birds fed *L. fermentum*-supplemented diet might benefit microbial homeostasis and alleviate inflammation. In a NE challenge model of broilers, *B. subtilis* DSM 32315 decreased the *Lachnospiraceae* members in the cecum (15), this was consistent with our findings that both *L. fermentum* and *B. coagulans* reduced the cecal *Lachnospiraceae* abundance. *Ruminococcus* spp. could produce lantibiotics that enhanced sterilization activity against some clostridia and bifidobacteria species (40), and the increase of *Ruminococcus* spp. was associated with mucin glycans (41). Xu et al. (34) reported that higher *Ruminococcus* spp. and *Bacteroides* spp. abundance was observed in the ileum of NE birds, which was decreased by *B. licheniformis* supplementation. Dietary *L. fermentum* decreased *Ruminococcus* spp. in the cecum, but *B. coagulans* increased *Bacteroides* spp. in the ileum of *C. perfringens*-challenged birds. *Bacteroides* spp. can evolve into a pathogenic form and will increase when the gut is pathologically impaired (42). In the current study, *L. fermentum* significantly increased *Lactobacillus* spp. in the ileum on day 21 and decreased *Faecalibacterium* spp. in the cecum on day 28. *Lactobacillus* spp. is a well-known beneficial genus in the gut and functions by maintaining intestinal bacterial community, improving immunity and gut integrity, and facilitating nutrition absorption (43). Complying with our findings, Li et al. (13) reported that *L. acidophilus* supplementation increased *Lactobacillus* spp. and suppressed *Faecalibacterium* spp., a potentially pathogenic flora.

*Peptostreptococcaceae* was one of the dominant families in the ileum of broilers (13, 32). In a broiler NE model established by *Eimeria maxima* and *C. perfringens* co-infection, challenged birds had higher ileal *Peptostreptococcaceae* and *Streptococcus* spp. abundance as compared to counterparts fed *Bacillus* direct-fed microbials (44). Similarly, *L. fermentum* decreased unclassified f_*Peptostreptococcaceae* in the ileum of *C. perfringens*-challenged birds on both days 21 and 28. However, *B. coagulans* increased *Streptococcus* spp. in the ileum on day 28. It has been indicated that there was a positive correlation between the relative abundance of *Escherichia-Shigella* with NE occurrence in broilers (45). Numerous studies evidenced that *C. perfringens* challenge increased the *Escherichia-Shigella* abundance in the ileum, and probiotic supplementation significantly or numerically decreased it (13, 25). However, in the present study, *C. perfringens* challenge decreased *Escherichia-Shigella* in the cecum on day 28, which was increased by *B. coagulans*. These data suggested that *B. coagulans* exerted a negative effect on intestinal microbiota at 7 days post challenge.

Membrane transport, carbohydrate metabolism, amino acid metabolism, replication and repair, and energy metabolism were the dominant functions of microbiota, which was confirmed by Zhang et al. (19). Membrane transport pathways are essential to cell viability and growth and are thereby crucial for the survival of bacteria in the gut ecosystem (46). Zhang et al. (19) reported that *C. perfringens* challenge decreased the abundance of the membrane transport function of ileal microbiota. In contrast, we found *C. perfringens* infection increased the membrane transport of cecal microbiota on day 21, which was decreased by probiotic addition. Meanwhile, challenged birds fed *B. coagulans*-supplemented diet had higher membrane transport abundance in the ileum on day 28. This indicated that the activities of ileal and cecal microbiota showed different responses to challenge and probiotic addition. It has been demonstrated that decreased carbohydrate metabolism and increased amino acid metabolism was observed in inflamed mucosal microbiota of ulcerative colitis patients (47). In the present study, *C. perfringens* challenge increased amino acid metabolism of ileal microbiota, but decreased it in cecal microbiota on day 21, which were conversely changed by probiotic addition. This might be attributed to the mucolytic characteristic and ileal colonization of *C. perfringens* (48). Dietary *L. fermentum* and *B. coagulans* affected the bacterial function by changing the microbial composition. Furthermore, challenged birds supplemented with *L. fermentum* had higher abundance of the replication and repair of ileal and cecal microbiota as well as translation of ileal microbiota on day 21. Consistently, Cui et al. (49) reported that dietary addition with *B. subtilis* and *Clostridium butyricum* increased the replication and repair of jejunal microbiota in broilers, which improved the renewal and vigorous vitality of bacteria.

## Conclusion

*C. perfringens* challenge induced inflammatory responses and intestinal microbial dysbiosis soon after challenge and these impairments diminished at 7 days post challenge. Dietary *L. fermentum* and *B. coagulans* supplementation attenuated inflammation by modulating Th cell function and T cell responses, and restored intestinal microflora by enriching beneficial bacteria and suppressing harmful flora. *L. fermentum* exhibited superior microbial modulatory effects to *B. coagulans*.

## Data Availability Statement

The sequencing data presented in the study are deposited in the Sequence Read Archive of the NCBI, accession number SPR304801.

## Ethics Statement

The animal study was reviewed and approved by the Institutional Animal Care and Use Committee of Wuhan Polytechnic University.

## Author Contributions

SG, YiX, and BD conceived and designed the experiment. SG, YiX, and TW performed the experiment. SG, YuX, TW, and DZ analyzed the data. SG, YuX, ZZ, and BD wrote the manuscript. All authors read and approved the final manuscript.

## Conflict of Interest

The authors declare that the research was conducted in the absence of any commercial or financial relationships that could be construed as a potential conflict of interest.
