# Supplementary material for: Dietary Lactobacillus fermentum and Bacillus coagulans Supplementation Modulates Intestinal Immunity and Microbiota of Broiler Chickens Challenged by Clostridium perfringens
Source: Front Vet Sci. 2021 May 31;8:680742. doi: 10.3389/fvets.2021.680742 (PMC8200825; doi:10.3389/fvets.2021.680742)
Supplement: Supplementary file 1 [file Data_Sheet_1.docx]

Supplementary Material

1. **Supplementary Figures and Tables**
   1. **Supplementary Figures**

**Figure S1. The CD3^+^ T cell distribution in jejunal mucosa determined by immunohistochemical analysis.** The picture was captured at 100 × magnification. The black arrows indicated the CD3^+^ T cells.

**Figure S2. The rarefaction curve analysis of ileal and cecal microbiota on days 21 (A, C) and 28 (B, D).** Data were derived from 8 and 7 chickens on days 21 and 28, respectively. Control, unchallenged group; Cp, *Clostridium perfringens*-challenged group; Lf_Cp, challenged group with dietary supplementation of *Lactobacillus fermentum*; Bc_Cp, challenged group with dietary supplementation of *Bacillus coagulans*.

**Figure S3. Veen diagram of the OTUs from ileal and cecal microbiota on days 21 (A, C) and 28 (B, D).** Data were derived from 8 and 7 chickens on days 21 and 28, respectively. Control, unchallenged group; Cp, *Clostridium perfringens*-challenged group; Lf_Cp, challenged group with dietary supplementation of *Lactobacillus fermentum*; Bc_Cp, challenged group with dietary supplementation of *Bacillus coagulans*.

**Figure S4. Relative abundances of bacteria at the phylum level in ileal and cecal microbiota on days 21 (A, C) and 28 (B, D).** Data were derived from 8 and 7 chickens on days 21 and 28, respectively. Control, unchallenged group; Cp, *Clostridium perfringens*-challenged group; Lf_Cp, challenged group with dietary supplementation of *Lactobacillus fermentum*; Bc_Cp, challenged group with dietary supplementation of *Bacillus coagulans*.

- 1. **Supplementary Tables**

**Table S1** The composition and nutrient levels of basal diet

| Items (%, unless otherwise indicated) | Starter diets (d 0 to 21) | Grower diets  (d 22 to 28) |
| --- | --- | --- |
| Ingredients |  |  |
| Barly | 26.00 | 10.00 |
| Corn | 25.00 | 44.44 |
| Soybean meal | 33.79 | 29.80 |
| Soybean oil | 6.28 | 7.49 |
| Fish sparerib meal | 5.00 | 5.00 |
| Dicalcium phosphate | 1.19 | 0.55 |
| Limestone | 1.14 | 1.30 |
| Sodium chloride | 0.35 | 0.35 |
| DL-Methionine (98%) | 0.25 | 0.12 |
| Choline chloride (50%) | 0.25 | 0.20 |
| Vitamin premix^1^ | 0.05 | 0.05 |
| Trance mineral premix^2^ | 0.20 | 0.20 |
| Zeolite powder | 0.50 | 0.50 |
| Calculated nutrient levels |  |  |
| Metabolic energy (Mcal/kg) | 2.95 | 3.15 |
| Crude protein | 21.50 | 19.50 |
| Calcium | 1.00 | 0.90 |
| Available Phosphorus | 0.45 | 0.35 |
| Lysine | 1.21 | 1.09 |
| Methionine | 0.59 | 0.43 |
| Threonine | 0.83 | 0.75 |

^1^The vitamin premix supplied the following per kilogram of diet: vitamin A, 12,500 IU; vitamin D_3_, 2,500 IU; vitamin K_3_ 2.65 mg; vitamin E, 30 IU; vitamin B_1_, 2 mg; vitamin B_2_, 6 mg; vitamin B_12_, 0.025 mg; biotin, 0.0325 mg; folic acid, 1.25 mg; pantothenic acid, 12 mg; nicotinic acid, 50 mg.

^2^The trace mineral premix supplied the following per kilogram of diet: copper, 8 mg; iron, 80 mg; zinc, 75 mg; manganese, 100 mg; selenium, 0.15 mg; iodine, 0.35 mg.

**Table S2** Primers used for quantitative real-time PCR.

| Gene name | Accession number | Forward sequence (5’-3’) | Reverse sequence (5’-3’) |
| --- | --- | --- | --- |
| β-actin | NM_205518 | GAGAAATTGTGCGTGACATCA | CCTGAACCTCTCATTGCCA |
| IL-1β | NM_204524 | ACTGGGCATCAAGGGCTA | GGTAGAAGATGAAGCGGGTC |
| IFN-γ | Y07922 | AGCTGACGGTGGACCTATTATT | GGCTTTGCGCTGGATTC |
| IL-13 | AJ621735 | CCAGGGCATCCAGAAGC | CAGTGCCGGCAAGAAGTT |
| IL-17 | AJ493595 | CTCCGATCCCTTATTCTCCTC | AAGCGGTTGTGGTCCTCAT |
| TGF-β4 | M31160 | CGGGACGGATGAGAAGAAC | CGGCCCACGTAGTAAATGAT |

**Table S3** Sequencing depth of samples from ileal and cecal contents at 21 days of age

| Sample name | Ileum | | |  | Cecum | | |
| --- | --- | --- | --- | --- | --- | --- | --- |
|  | Numbers of sequences | Average length (bp) | Good’s coverage (%) |  | Numbers of sequences | Average length (bp) | Good’s coverage (%) |
| Control1 | 118,838 | 458 | 99.8 |  | 114,855 | 450 | 99.3 |
| Control2 | 28,633 | 457 | 99.9 |  | 113,674 | 449 | 99.5 |
| Control3 | 95,974 | 457 | 99.9 |  | 90,960 | 450 | 99.4 |
| Control4 | 93,146 | 453 | 99.8 |  | 68,742 | 450 | 99.5 |
| Control5 | 100,736 | 454 | 99.9 |  | 98,449 | 449 | 99.4 |
| Control6 | 96,773 | 444 | 99.8 |  | 111,818 | 448 | 99.5 |
| Control7 | 106,122 | 443 | 99.8 |  | 217,472 | 449 | 99.4 |
| Control8 | 99,185 | 449 | 99.8 |  | 56,240 | 447 | 99.4 |
| Cp1 | 92,393 | 444 | 99.9 |  | 252,684 | 451 | 99.5 |
| Cp2 | 132,881 | 454 | 99.9 |  | 154,594 | 446 | 99.5 |
| Cp3 | 56,349 | 441 | 99.8 |  | 189,534 | 448 | 99.5 |
| Cp4 | 125,518 | 441 | 99.9 |  | 208,594 | 448 | 99.4 |
| Cp5 | 176,052 | 447 | 99.8 |  | 156,276 | 447 | 99.4 |
| Cp6 | 164,036 | 440 | 99.9 |  | 105,507 | 448 | 99.3 |
| Cp7 | 153,543 | 440 | 99.9 |  | 151,219 | 447 | 99.5 |
| Cp8 | 58,367 | 442 | 99.9 |  | 260,633 | 449 | 99.5 |
| Lf_Cp1 | 29,707 | 454 | 99.9 |  | 159,652 | 451 | 99.5 |
| Lf_Cp2 | 94,194 | 464 | 99.9 |  | 116,778 | 448 | 99.5 |
| Lf_Cp3 | 117,068 | 464 | 99.9 |  | 202,787 | 450 | 99.5 |
| Lf_Cp4 | 151,639 | 462 | 99.9 |  | 198,441 | 450 | 99.3 |
| Lf_Cp5 | 54,310 | 462 | 99.8 |  | 188,472 | 445 | 99.4 |
| Lf_Cp6 | 125,212 | 451 | 99.7 |  | 194,600 | 451 | 99.4 |
| Lf_Cp7 | 153,019 | 452 | 99.9 |  | 124,283 | 443 | 99.4 |
| Lf_Cp8 | 111,904 | 463 | 99.8 |  | 84,745 | 449 | 99.4 |
| Bc_Cp1 | 121,187 | 443 | 99.8 |  | 269,877 | 448 | 99.6 |
| Bc_Cp2 | 113,072 | 441 | 99.9 |  | 228,769 | 445 | 99.4 |
| Bc_Cp3 | 42,538 | 441 | 99.9 |  | 173,128 | 448 | 99.5 |
| Bc_Cp4 | 95,725 | 453 | 99.9 |  | 102,325 | 451 | 99.5 |
| Bc_Cp5 | 107,540 | 459 | 99.9 |  | 127,670 | 451 | 99.4 |
| Bc_Cp6 | 125,134 | 443 | 99.7 |  | 13,179 | 447 | 99.6 |
| Bc_Cp7 | 41,790 | 458 | 99.9 |  | 195,346 | 446 | 99.4 |
| Bc_Cp8 | 33,017 | 457 | 99.9 |  | 144,875 | 450 | 99.4 |

**Table S4** Sequencing depth of samples from ileal and cecal contents at 28 days of age

| Sample name | Ileum | | |  | Cecum | | |
| --- | --- | --- | --- | --- | --- | --- | --- |
|  | Numbers of sequences | Average length (bp) | Good’s coverage (%) |  | Numbers of sequences | Average length (bp) | Good’s coverage (%) |
| Control1 | 71,063 | 426 | 99.9 |  | 42,239 | 434 | 99.8 |
| Control2 | 55,787 | 444 | 99.8 |  | 59,557 | 434 | 99.9 |
| Control3 | 52,037 | 437 | 99.8 |  | 61,824 | 433 | 99.9 |
| Control4 | 54,683 | 432 | 99.9 |  | 53,490 | 432 | 99.8 |
| Control5 | 50,982 | 428 | 99.8 |  | 64,383 | 431 | 99.9 |
| Control6 | 60,360 | 429 | 99.8 |  | 53,519 | 439 | 99.8 |
| Control7 | 60,140 | 425 | 99.9 |  | 58,442 | 433 | 99.9 |
| Cp1 | 58,231 | 432 | 99.9 |  | 57,181 | 432 | 99.9 |
| Cp2 | 31,273 | 432 | 99.9 |  | 53,171 | 433 | 99.9 |
| Cp3 | 39,592 | 427 | 99.8 |  | 51,071 | 429 | 99.9 |
| Cp4 | 51,238 | 428 | 99.9 |  | 52,363 | 430 | 99.9 |
| Cp5 | 42,450 | 429 | 99.8 |  | 62,677 | 433 | 99.9 |
| Cp6 | 63,495 | 425 | 99.8 |  | 59,870 | 437 | 99.8 |
| Cp7 | 38,513 | 426 | 99.8 |  | 55,227 | 431 | 99.8 |
| Lf_Cp1 | 42,719 | 438 | 99.8 |  | 71,632 | 430 | 99.9 |
| Lf_Cp2 | 72,177 | 440 | 99.9 |  | 63,778 | 435 | 99.8 |
| Lf_Cp3 | 53,979 | 430 | 99.8 |  | 58,723 | 431 | 99.9 |
| Lf_Cp4 | 62,432 | 438 | 100.0 |  | 63,992 | 430 | 99.9 |
| Lf_Cp5 | 46,803 | 441 | 99.9 |  | 53,437 | 434 | 99.9 |
| Lf_Cp6 | 50,233 | 440 | 99.9 |  | 45,763 | 437 | 99.8 |
| Lf_Cp7 | 48,604 | 434 | 99.9 |  | 59,893 | 435 | 99.8 |
| Bc_Cp1 | 61,718 | 442 | 99.9 |  | 74,927 | 433 | 99.8 |
| Bc_Cp2 | 55,765 | 446 | 99.9 |  | 52,637 | 435 | 99.9 |
| Bc_Cp3 | 50,931 | 434 | 99.9 |  | 53,545 | 431 | 99.9 |
| Bc_Cp4 | 44,288 | 432 | 99.9 |  | 58,717 | 431 | 99.8 |
| Bc_Cp5 | 73,447 | 439 | 99.9 |  | 65,839 | 431 | 99.9 |
| Bc_Cp6 | 35,122 | 439 | 99.9 |  | 67,457 | 431 | 99.9 |
| Bc_Cp7 | 55,118 | 448 | 99.9 |  | 48,873 | 435 | 99.9 |
