# Supplementary figures and images for: Dietary Lactobacillus fermentum and Bacillus coagulans Supplementation Modulates Intestinal Immunity and Microbiota of Broiler Chickens Challenged by Clostridium perfringens
Source: Front Vet Sci. 2021 May 31;8:680742. doi: 10.3389/fvets.2021.680742 (PMC8200825; doi:10.3389/fvets.2021.680742)

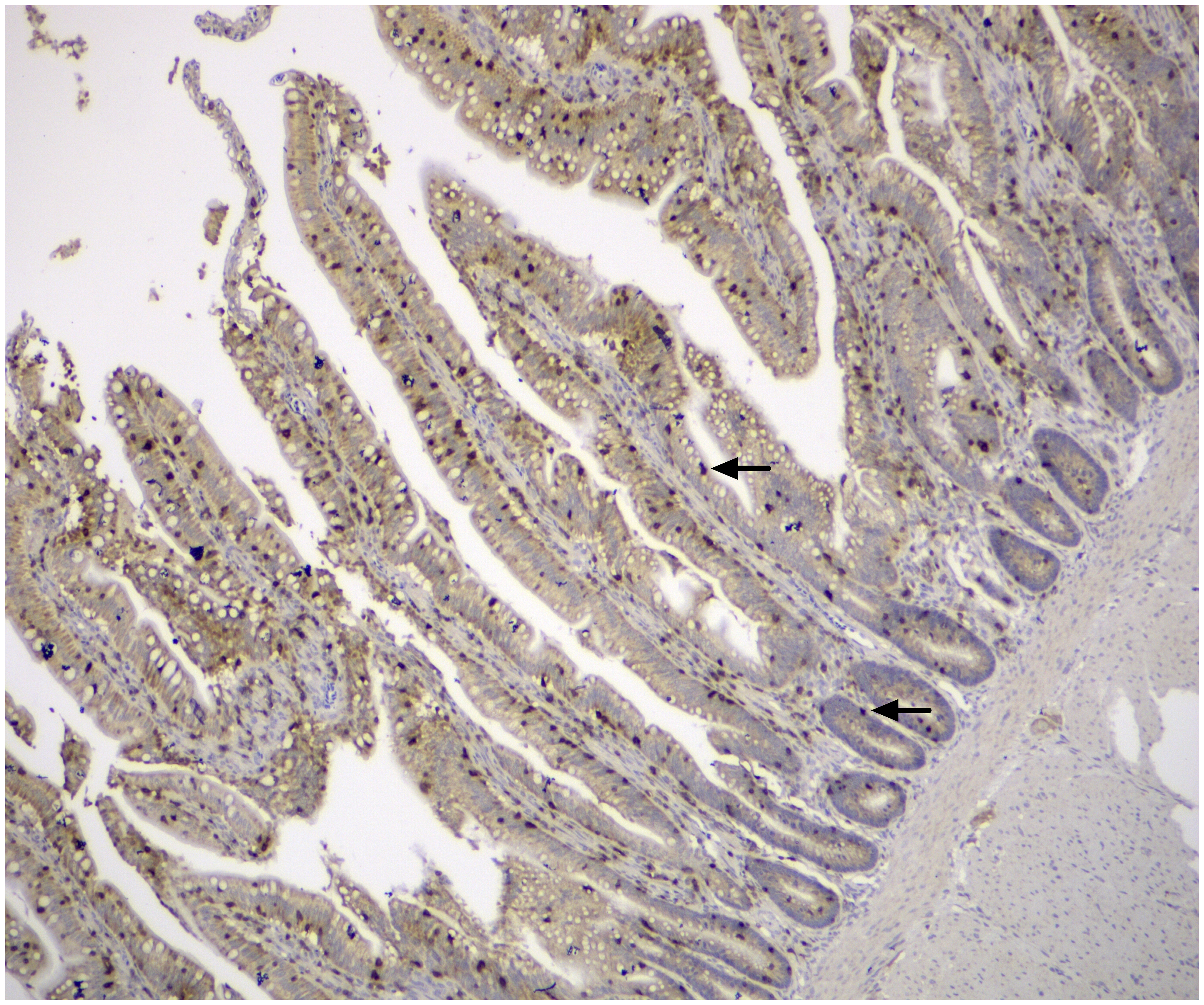

Supplement: Supplementary file 2 [file Image_1.TIFF]

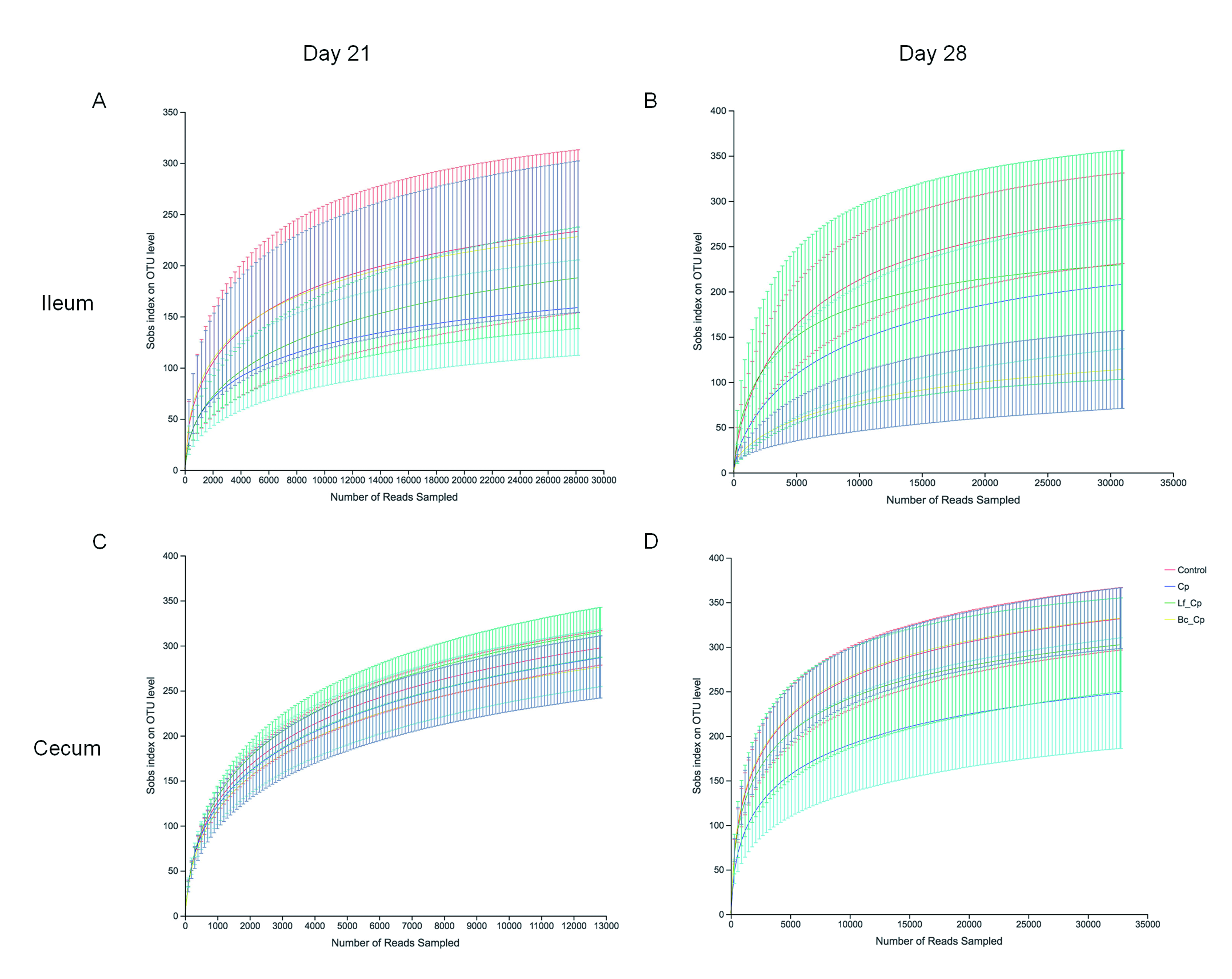

Supplement: Supplementary file 3 [file Image_2.TIF]

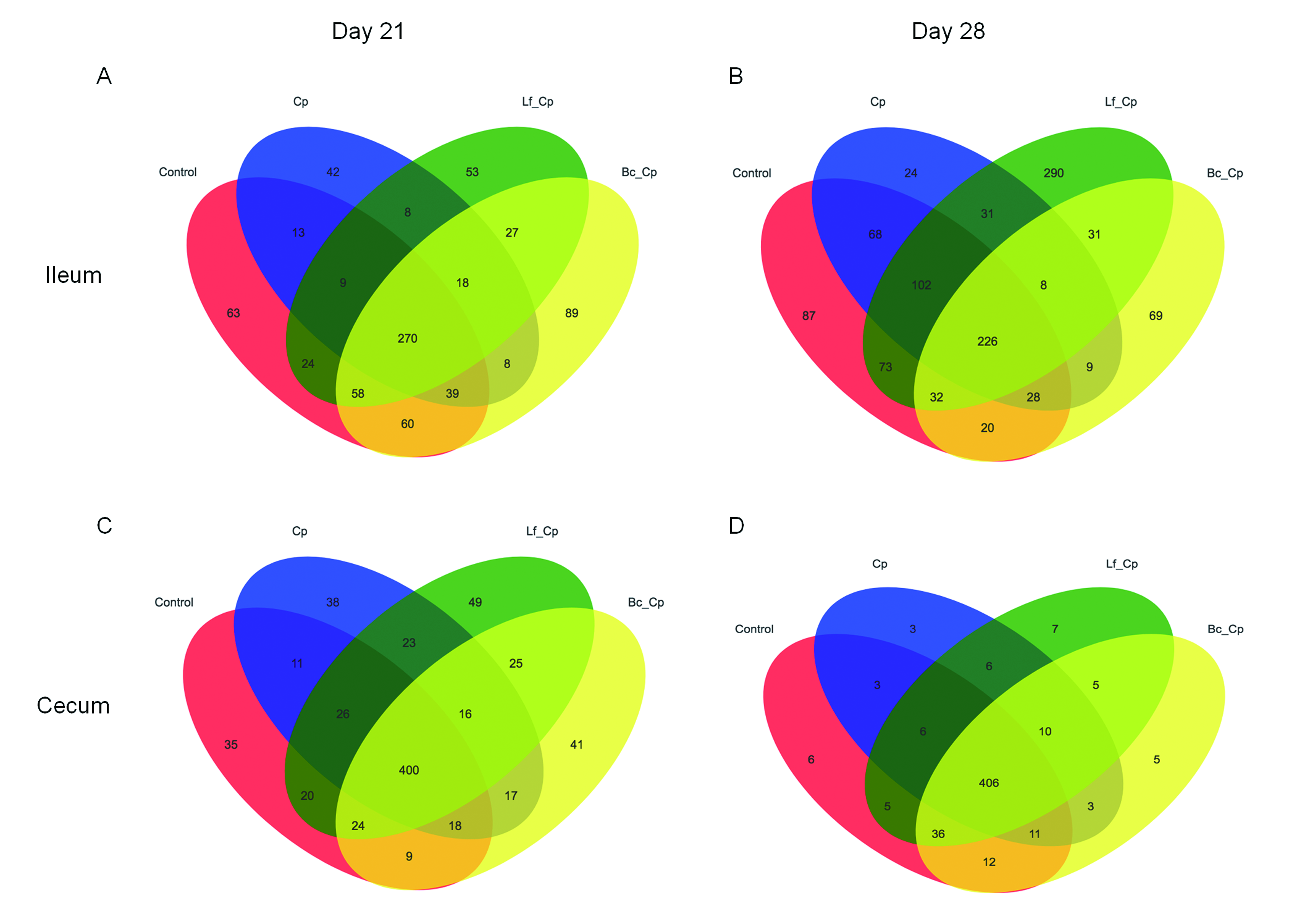

Supplement: Supplementary file 4 [file Image_3.TIF]

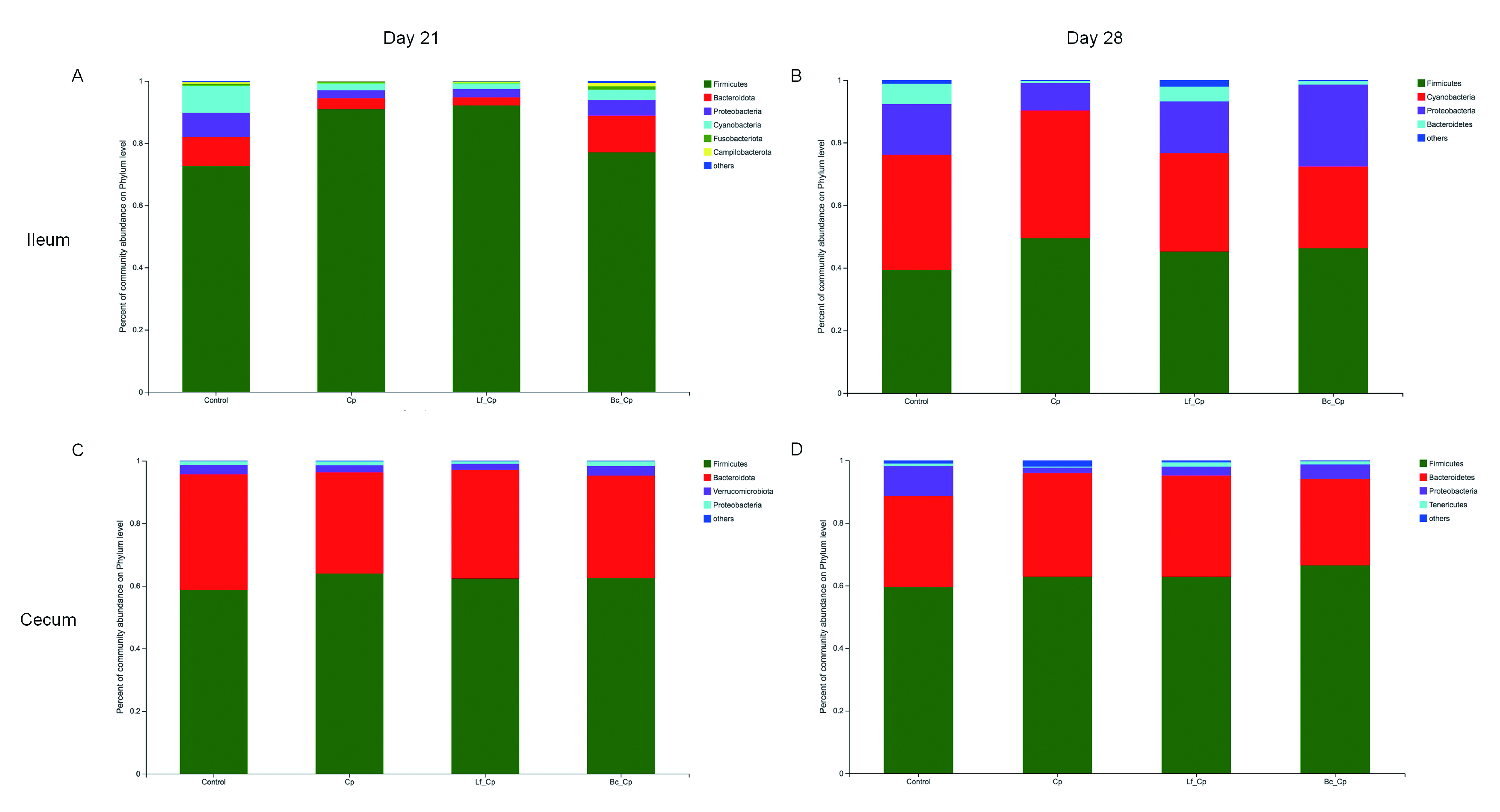

Supplement: Supplementary file 5 [file Image_4.TIF]
